# Holt-Oram Syndrome: An Incidental Diagnosis

**DOI:** 10.7759/cureus.24899

**Published:** 2022-05-11

**Authors:** Mehak Gupta, Ayodeji Dosu, Jayesh Makan

**Affiliations:** 1 Cardiology, Shrewsbury and Telford Hospital NHS Trust, Telford, GBR; 2 Internal Medicine, Shrewsbury and Telford Hospital NHS Trust, Telford, GBR

**Keywords:** atrial septal defect, tbx3 and tbx5 gene mutations, adult congenital heart disease (achd), oram, holt

## Abstract

Holt-Oram syndrome is a rare autosomal dominant disorder which occurs because of mutations in the TBX5 genes. Most notable manifestations include musculoskeletal deformities, predominantly affecting the upper limbs, and congenital heart defects. Presentation could be multifaceted leading to delay in diagnosis. We describe an interesting incidental diagnosis of Holt-Oram syndrome in a young female adult who accompanied her son to the clinic. He had undergone closure of both atrial septal defect (ASD) and patent ductus arteriosus (PDA) in his infancy. She reported progressive exertional dyspnoea, reduced exercise tolerance, and palpitations; incidentally, she was noted to have right upper limb deformities. These findings prompted further evaluation and thereafter, resulted in a diagnosis of Holt-Oram syndrome.

## Introduction

Holt-Oram syndrome or heart-hand syndrome consists of genotypic and phenotypic abnormalities. It is clinically characterized by a morphological deformity of the upper limbs, congenital cardiac defects, and/or conduction abnormalities [1]. It is an autosomal dominant disorder due to a mutation in TBX5 and TBX3 genes located on chromosome 12, but sporadic cases have also been reported [2]. It has an incidence of one in 100,000 liveborn infants [3] and few cases have been reported so far.

## Case presentation

We present the case of a 49-year-old female who reported symptoms of gradually worsening dyspnoea, reduced exercise tolerance, associated lethargy, and intermittent palpitations. Of note, her son underwent atrial septal defect (ASD) and patent ductus arteriosus (PDA) closure in his infancy and her grandfather passed away from a heart condition unknown to her. There was no other relevant past medical history. On examination, a soft ejection systolic murmur was auscultated in the pulmonary area with a normal second heart sound. An upper limb deformity was noted with an absent little finger, a finding not previously explained to her and assumed to be a congenital deformity.

**Figure 1:**
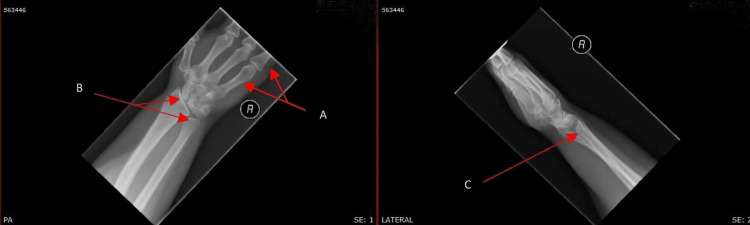
X-ray of the right wrist joint showing absent little finger metacarpal and proximal phalanx (arrow A). Distal radius and ulnar head are slightly dysplastic (arrow B). Diastasis at the distal radio-ulnar joint with negative ulnar variance was noted (arrow C).

**Figure 2:**
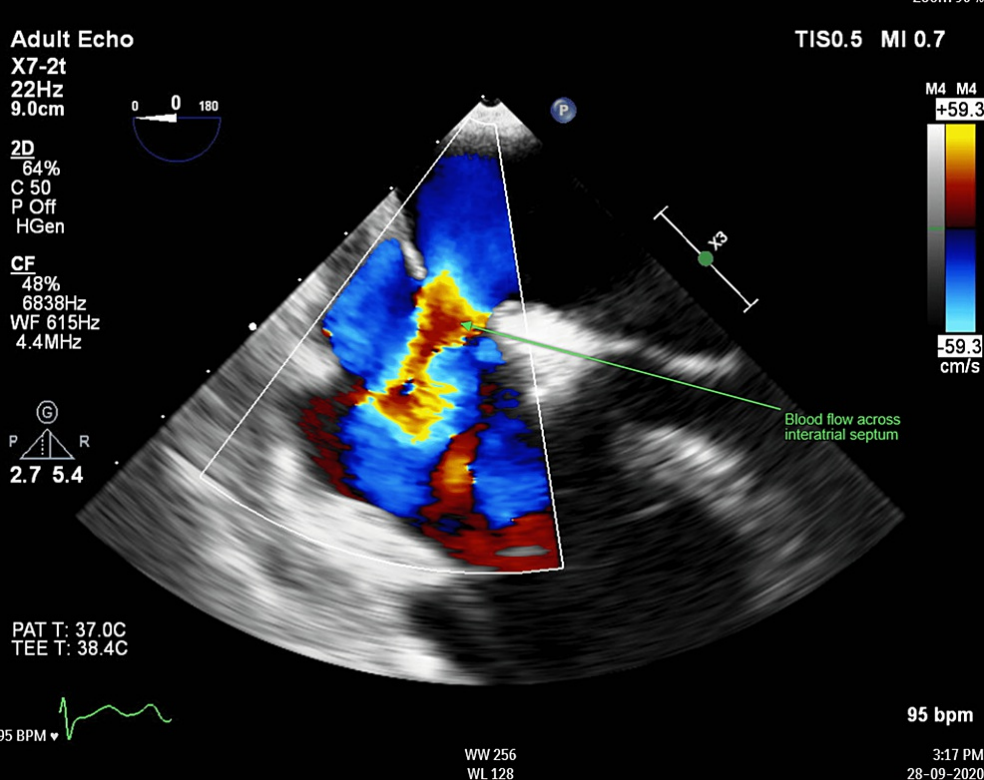
Transoesophageal echocardiogram demonstrating blood flow across the interatrial septum (green arrow)

A resting electrocardiogram (ECG) showed sinus rhythm with a partial right bundle branch block pattern; upper limb X-ray findings are shown in Figure 1. An echocardiogram performed showed left to right flow across the interatrial septum suggesting small secundum ASD with a dilated and volume-overloaded right ventricle. She proceeded to have a transoesophageal echocardiogram confirming the presence of a secundum ASD (Figure 2). At this point, a diagnosis of Holt-Oram syndrome was made. She was referred to a tertiary centre under the care of an adult congenital heart disease team, where she underwent transcatheter closure of the septal defect alongside being referred to a geneticist and hand occupational therapist.

## Discussion

First reported in 1960, Holt-Oram syndrome [4] is characterized by upper limb deformities varying from phocomelia to subtle abnormalities of the radial ray, such as a triphalangeal or bifid thumb and radial‐ulnar anomalies. In the majority of cases, the abnormalities are observed on the left side of the body as opposed to our patient [5]. Cardiac anomalies have also been noted in 75% of patients, ranging from simple atrial or ventricular septal defects (as observed in our patient) to the major left heart and conotruncal defects [6]. Various arrhythmias including conduction blocks can also be observed in isolation or in association with the above-mentioned congenital heart defects, with the most common being sinus bradycardia and right bundle branch block [7] as noted in our patient.

The underlying genetic defect in Holt-Oram syndrome is located on the long arm of chromosome 12 (12q2) wherein lie genes which play a vital role in cardiac and skeletal development [8], mutations within them give rise to a wide range of phenotypes typical of Holt-Oram syndrome. Mutations within the TBX3 and TBX5 genes provide an embryologic basis for the prevalence of atrial and/or ventricular septal defects in patients with Holt-Oram syndrome [9]. The TBX5 gene mutation is seen in 74% of clinically detected cases [10], but some sporadic cases have also been reported [2].

The management of Holt-Oram syndrome is individualised and based on symptomatology. A multidisciplinary team approach is used involving the cardiologist, cardiovascular surgeons, occupational therapists, physiotherapists, and orthopaedic surgeons. Echocardiograms should be done every one to five years in those with heart defects as well as an annual surveillance electrocardiogram to look for conduction abnormalities. Medications are used in patients with signs of heart failure, as well as treatment of arrhythmias and closure or repair of heart defects can be offered in symptomatic patients [11].

Genetic tests and counselling given to parents may ease decision-making regarding reproduction and/or in being more vigilant during pregnancy. If the diagnosis has been made in the parent, prenatal screening should be offered. Offspring of an affected individual are at a 50% risk of being affected [11]. Prenatal diagnostics play a vital role as early prenatal identification of congenital defects allows parents to establish whether to terminate the pregnancy or to adequately plan labour and ensure early neonatal specialist input [12]. Through genetic counselling, we can also ascertain the risk of repeating defects in the family, which is a vital element of primary prevention [13].

## Conclusions

This case report signifies that Holt-Oram syndrome should be considered in patients with cardiac symptoms and upper limb deformities with a personal or family history of heart defects, as the diagnosis was not suspected in our patient for several decades into adulthood. It is essential to make an early diagnosis as it gives the individual an opportunity to make conscientious decisions regarding reproduction and make use of prenatal screening for any congenital abnormalities. Genetic testing and counselling enable us to screen and diagnose other family members who may not yet be symptomatic.
